# Pulmonary Edema in Healthy Subjects in Extreme Conditions

**DOI:** 10.1155/2011/275857

**Published:** 2011-06-22

**Authors:** Erika Garbella, Giosuè Catapano, Lorenza Pratali, Alessandro Pingitore

**Affiliations:** ^1^Clinical Physiology Institute, CNR, Via Moruzzi 1, 56124 Pisa, Italy; ^2^Fondazione G. Monasterio, CNR, Regione Toscana, Via Moruzzi 1, 56124 Pisa, Italy; ^3^Extreme Center, Scuola Superiore S. Anna, Piazza Martiri della Libertà 33, 56127 Pisa, Italy

## Abstract

There are several pieces of evidence showing occurrence of pulmonary edema (PE) in healthy subjects in extreme conditions consisting of extreme psychophysical demand in normal environment and psychophysical performances in extreme environment. A combination of different mechanisms, such as mechanical, hemodynamic, biochemical, and hypoxemic ones, may underlie PE leading to an increase in lung vascular hydrostatic pressure and lung vascular permeability and/or a downregulation of the alveolar fluid reabsorption pathways. PE can be functionally detected by closing volume measurement and lung diffusing capacity test to different gases or directly visualized by multiple imaging techniques. Among them chest ultrasonography can detect and quantify the extravascular lung water, creating “comet-tail” ultrasound artefacts (ULCs) from water-thickened pulmonary interlobular septa. In this paper the physiopathological mechanisms of PE, the functional and imaging techniques applied to detect and quantify the phenomenon, and three models of extreme conditions, that is, ironman athletes, climbers and breath-hold divers, are described.

## 1. Introduction

**Figure 1:**
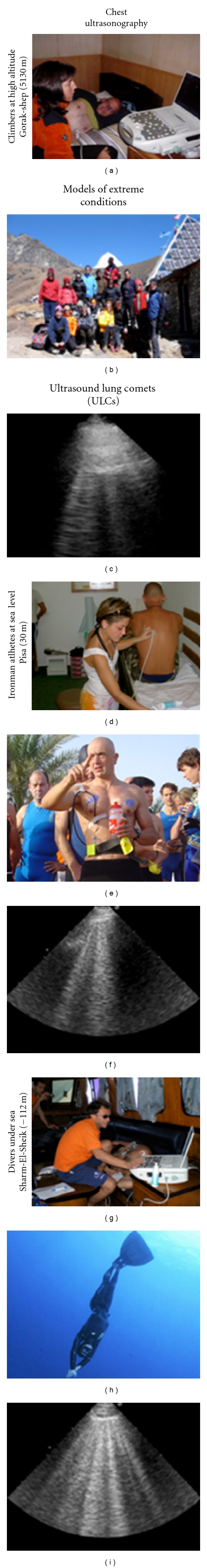
Three different models of extreme conditions: climbers at 5130 meters above sea level; ironman athletes at sea level; breath-hold diver at −112 meters under sea level. In all the above-mentioned conditions subjects were monitored by chest ultrasonography that showed the presence of ULCs.

Pulmonary edema (PE) is a life-threatening condition that may lead to acute respiratory failure. There are several pieces of evidence showing the occurrence of PE in healthy subjects in extreme environment and/or under extreme psychophysical stress [1–3]. Human extreme conditions can be considered in terms of extreme psychophysical demand in normal environment, such as for ironman athletes, or in terms of psychophysical performances in extreme environment, such as for climbers or breath-hold deep divers (Figure 1). Susceptibility to PE has been shown to be related to pulmonary hemodynamics and ventilatory drive (precisely reduced hypoxic ventilatory response), that lead to a greater hypoxic stimulus and/or a pulmonary hemodynamic hyperresponse to the above-mentioned extreme conditions. Finally, a combination of different and not well-defined yet mechanisms, such as mechanical, hemodynamic, biochemical, and hypoxemic ones, may underlie PE, leading to a greater increase in lung vascular hydrostatic pressure (either heterogeneous or not), an increase in lung vascular permeability, and/or a downregulation of the alveolar fluid reabsorption pathways.

PE can be functionally detected by an increase in closing lung volume (the volume at which small airways start closing), reflecting an early small airways compression due to pulmonary interstitial fluid accumulation [4]. Another functional method is the lung diffusing capacity test (DL) to different gases (carbon monoxide, CO and nitric oxide, NO), being expression of the alveolar-capillary membrane integrity [5]. Moreover PE can be directly visualized by multiple imaging techniques, such as chest radiography, computed tomography (CT), magnetic resonance (MR), and chest ultrasonography [2, 6]. Among these, chest ultrasonography detects and quantifies extravascular lung water, creating “comet-tail” ultrasound artefacts (ULCs) from water-thickened pulmonary interlobular septa [7]. This technique is suitable, versatile, not invasive, nonionizing and hand portable, a fundamental option when studying subjects directly on the field and not in a dedicated imaging laboratory. 

In this paper the physiopathological mechanisms of PE, the functional and imaging techniques applied to detect and quantify the phenomenon, and three models of extreme conditions, that is, ironman athletes, climbers and breath-hold divers, are described.

## 2. Physiopathology

The pulmonary blood-gas barrier is continuously subject to mechanical stress. Precisely its integrity depends on balance between circumferential tension (related to the capillary transmural pressure and the radius of capillary's curvature), longitudinal tension in the alveolar wall elements (related to the inflation of the lung), and surface tension of the alveolar lining layer [8, 9]. Therefore, “stress failure” describes mechanically induced breaks in the blood-gas barrier. In the last years, West and others have reported a great deal of experimental data describing the effects of mechanical stress on the lung [10–12]. Vulnerability to pulmonary capillary stress failure varies between species and increases at high lung inflation [13]. Exercise increases the potential risk of pulmonary capillary stress failure. In fact increase in pulmonary arterial pressure together with active expiration and increased lung inflation resulting from it exert mechanical forces on the pulmonary microvasculature. Moreover pulmonary capillary stress failure is associated with an increased permeability to protein and red blood cells, as shown by their increased concentration in the bronchoalveolar lavage fluid [14]. Another physiological mechanism that potentially augments pulmonary capillary pressure is the uneven vasoconstriction of the pulmonary arteries, resulting in heterogeneous distribution of the blood flow within the vascular bed and thus in a regional capillaries' overperfusion (i.e., in areas with the least arterial vasoconstriction) [15, 16]. Moreover, once the drop of alveolar oxygen tension is detected by oxygen sensors located in the pulmonary vasculature, it follows vasoconstriction of both small pulmonary arteries and pulmonary veins [17–19]. Physiologically, inhibition of voltage-dependent potassium channels, membrane depolarization, and calcium entry through L-type calcium channels are involved in the response to acute hypoxia of smooth-muscle cells in the pulmonary vasculature, that begins within few seconds [18, 20]. However it remains to be determined if constitutively decreased mRNA expression or an acquired transcriptional defect of voltage-dependent potassium channels protein is at the origin of oedema susceptibility. On the other hand the regional hypoxic vasoconstriction, that leads to a nonhomogeneous distribution of pulmonary blood flow, thus possibly to lung edema, could be explained either by an uneven distribution of alveolar ventilation or a heterogeneous oxygen sensing within pulmonary vasculature smooth muscle cells [21–24]. Furthermore, also a constitutionally pulmonary vascular hyperreactivity to hypoxia seems to have an important role behind lung edema. In fact hypoxia has been shown to impair, in predisposed subjects, systemic endothelial function, resulting in decreased bioavailability of NO and its second messenger cGMP, that is likely to contribute to the enhanced hypoxic pulmonary vasoconstriction [14, 25, 26]. Moreover other factors may be involved in vascular hyperreactivity to hypoxia, such as a sustained elevation of cytoplasmic calcium concentration, sympathetic activity, hypersecretion in the pulmonary circulation of vasoconstrictor mediators (e-selectin, endothelin-1), and reduced activity of protective enzymatic products of hemooxigenase-1 (inhibitors of hypoxia-induced vasoconstrictive and proinflammatory pathways) [27–31]. Finally another factor that may be involved in alveolar fluid accumulation and PE severity is a downregulation of the sodium-water transport pathway that leads to reduction in net alveolar fluid reabsorption [32].

Besides the local pulmonary mechanisms of PE, systemic factors may also predispose to PE. Exercise-associated hyponatremia, due to altered body fluid homeostasis, represents emerging potential cause of both PE and cerebral edema [33]. Several mechanisms have been considered. Hyponatremia may be the consequence of either sodium depletion, as in salty sweaters without adequate salt intake, or sodium dilution, secondary to excessive water intake [34, 35]. Interestingly a variant of the syndrome of inappropriate antidiuretic hormone secretion, with a hypersecretion of arginine vasopressin, has also been implicated as a mechanism of exercise-associated hyponatremia [36]. Sodium depletion would lead to hypoosmolar hyponatremia accompanied by hypovolemia and urine concentration, whereas dilutional hyponatremia is accompanied by euvolemia or hypervolemia and dilute urine. Moreover, the above-mentioned dilutional hyponatremia contrasts with the one mediated by inappropriate arginine vasopresson secretion, that is characterized by less than maximally diluite urine, natriuresis marked by a urinary Na concentration, and measurable plasma levels of arginine vasopressin [35]. Further a nonosmotic stimulation of arginine vasopressin is the enhanced release of muscle-derived interleukin (IL) 6 during glycogen depletion, as documented by administration of recombinant IL-6 in healthy subjects [37]. 

Perivascular inflammatory cell infiltration as well as increased serum levels of proinflammatory IL-1*β* and IL-6 has been reported in clinical cases of primary pulmonary hypertension but it is not fully clear up to now the role of pulmonary inflammation in the pathogenesis of acute PE in healthy subjects under extreme psychophysical conditions. Exhaustive endurance exercise leads to systemic inflammatory response secondary to metabolic, hormonal, thermal stress, and muscle damage thus potentially affecting microvascular endothelial function and lung tissue integrity. Moreover, in endurance athletes the activation of the inflammatory cascade within six hours from the end of strenuous exercise has been associated with a reduction in ventilatory performance [38].

## 3. Pulmonary Function Tests

It is well known that prolonged heavy exercise increases lung ventilo-perfusive heterogeneity and affects the efficiency of gas exchange [21, 39]. The effect of repeated exercise on pulmonary gas exchange has been used to elucidate the importance of structural versus functional mechanisms as a cause for the gas exchange impairment during exercise. Several authors have suggested that sustained exercise of maximum or near maximum effort might result in a structural injury (stress failure) of the alveolar-capillary membrane, likely when pulmonary arterial pressures is over 40 mmHg [10, 11, 39, 40]. Moreover stress failure may lead to increased fluid permeability and to edema. Finally, PE seems to worsen gas exchange in terms of arterial blood oxygen content (decrease in oxygen partial pressure and/or haemoglobin saturation), only at advanced stages, that is during alveolar edema [41]. Instead, early subclinical phases of interstitial edema can be functionally detected by increase in closing volume and/or reduction in DLCO [4, 5]. Indeed DLCO reflects the alveolar-capillary membrane integrity condition, being the expression of the CO passage through the alveolar-capillary membrane and into the plasma and the erythrocytes, as defined by the Roughton and Forster equation. DLCO is thus expected to be persistently reduced in case of thickening of the alveolar-capillary wall (interstitial oedema), while temporarily increased during blood shift and persistently and consistently increased in case of alveolar haemorrhage [42]. Moreover, DLNO is thought to represent the true membrane diffusing capacity as it is not dependent from pulmonary capillary blood volume and flow because of its very high affinity for haemoglobin. Therefore, DLNO/DLCO ratio can be used to differentiate between thickened alveolocapillary membrane (DLNO and DLCO are both decreased; DLNO/DLCO ratio is normal) and decreased perfusion of ventilated alveoli (DLNO less decreased than DLCO, DLNO/DLCO ratio is high) showing the presence of ventilo-perfusive mismatch [5]. Finally the rationale for measuring closing volume to detect early PE is that increased pulmonary extravascular fluid would be expected to early compress airways and thus increase the volume at which small airways close [43]. The method based on Guy's single breath techniques, that does not require foreign inert gases or 100% of oxygen, can be used to measure closing volume with hand-portable equipment directly at the place of the extreme performance [44].

## 4. Imaging Techniques

Radiographic, CT, or MRI imaging resulted equally effective in detecting PE induced by exhaustive endurance sports [2]. Since increased blood flow after exercise may be misdiagnosed as edema, time elapse of at least 30 minutes between the end of exercise and the postexercise imaging has been suggested to allow normalization of pulmonary capillary blood flow and volume [45]. Despite a large range time of PE evaluation after exercise (from 2 minutes to 2 hours), no differences in the frequency of edema have been reported suggesting that PE occurs during and not following exercise [2]. However, since the lung has been shown to recover rapidly from hydrostatic-induced pulmonary damage, that is, when pulmonary capillary transmural pressure backs to normal, the incidence of edema formation may be underestimated by imaging techniques and it may be difficult to be quantified after exercise. By the way, in early stage of high-altitude PE (HAPE) chest radiographs showed a patchy, peripheral distribution of edema that becomes more homogeneous and diffuses in advanced cases and during recovery [46]. 

Immersion PE has been seen in the dependent lung on CT scans (in special forces combat swimmers at the lateral decubitus position and in elite breath-hold divers at the level of the superior as well as parahilar zones bilaterally) whereas recurrent HAPE has showed patchy distribution of pulmonary infiltrates bilaterally on chest radiographs and on CT scans. These last findings strongly support the heterogeneous distribution of elevated capillary pressures, that in summary is likely to rely on an unevenly distributed hypoxic vasoconstriction in either pulmonary arteries or veins, or both.

The technical developments and the use of high-field-strength MR have improved the image quality, with a better signal-to-noise ratio, and the speed of acquisition of MR imaging, overwhelming the motion artefact due to heart and lung movements and susceptibility due to the presence of air in the lung. MR can give in the same exam information regarding both tissue characterization (in this context the presence of pulmonary fluid accumulation) and perfusion. In the clinical practice lung MR has comparable sensitivity to CT for the detection of lung diseases with MR having lower spatial but greater contrast resolution than CT [2, 47]. MR arterial spin labeling measures of pulmonary perfusion have been used to detect the ventilo-perfusive mismatch, that is known to increase under strenuous exercise, relating spatial perfusion heterogeneity to the exercise-induced changes in ventilation distribution. When exposed to normobaric hypoxia, HAPE-susceptible subjects have showed a more heterogeneous regional pulmonary blood flow than individuals without HAPE. These novel data suggest that uneven hypoxic pulmonary vasoconstriction is an important feature of subjects developing HAPE [16]. Despite the high accuracy of CT and MR in detecting PE, both techniques suffer of limitations consisting of high costs, low availability, exam complexity, duration, and, only for CT, ionizing exposure, that make them not suitable for studying healthy subjects on the field. Differently, chest ultrasonography is a highly performable imaging technique for its low time exam consuming, low cost, high versatility and availability. The “ultrasound comet-tail image” is an echographic image detectable by a cardiac ultrasound probe positioned over the chest. This image consists of multiple comet tails fanning out from the lung surface. They originate from water-thickened interlobular septa, and they give quantification of the extravascular lung water excess. They also provide an indirect measurement of pulmonary wedge pressure and a sensitive and accurate detection of even early subclinical interstitial edema. In a pig model of oleic-acid-induced lung injury, that mimics human Acute Respiratory Distress Syndrome, ULCs unmasked accumulation of extravascular lung water, then verified histologically, very early in the course of lung injury, even at a stage when no changes in hemogasanalytic parameters could be observed [48]. In clinical practice there is a great potential for this hand-portable technology that allows to quantify lung edema in real time, noninvasively, and with a radiation-free method [49]. In a head-to-head simultaneous comparison study of chest radiographs and ULCs, a linear correlation between echocardiographic comet score and radiologic lung water score (*r* = .78; *P* < .01) was found [6]. Further, ULCs have been proven useful in the differential diagnosis of dyspnoea of uncertain cause at the Emergency Room and for risk stratification in patients with heart failure or admitted with acute coronary syndrome [50–53]. The technique requires ultrasound scanning of the anterior right and left chest, from the second to the fifth intercostal space. It is simple (with a learning curve of less than 10 examinations) and fast to perform (requiring less than 3 minutes), and it is independent of the cardiac acoustic window. It requires very basic 2D technology imaging, even without a second harmonic or Doppler. The great advantage of this method is the versatility that allows to study free divers and climbers in their natural home, the sea and the mountains.

## 5. Extreme Psychophysical Performance in Normal Environment

Ironman race is also called ultratriathlon and consists of the following three different endurance sports: swim (2.4 miles, 3.86 km), bike ride (112 miles, 180 km), and marathon (26.2 miles, 42.2 km). This multidisciplinary sport demands an extraordinary psicophysical performance in terms of both endurance and exercise intensity. Reversible changes in the cardiovascular system, consisting of biohumoral and functional signs of cardiac damage have been documented after an ironman race. In particular there is an increase in cardiac troponins and B-type natriuretic peptide in presence of impairment of regional and global cardiac performance [54]. Indeed exhaustive endurance exercise induces ultra-structural damage of muscle tissue and release of metabolic, hormonal, thermal, and oxidative stress factors, which can give rise to systemic inflammatory and immune system response [55, 56]. Particularly after a triathlon race there is evidence of augmented proinflammatory interleukins (such as IL-8, IL-6, TNF-alpha, IL-1B), that are also markers of increased vascular permeability and bronchial inflammation [57–59].

The likelihood of developing clinical PE is at least 65% in presence of maximum or near-maximum effort, whereas it is low or absent in presence of submaximal exercise. In this context interstitial PE has been detected by various imaging techniques, such as TC scan, MR, chest ultrasonography, as well as by a decline in lung function (particularly of vital capacity and flow rates at mid and low lung volumes) and impairment of the pulmonary blood gas-barrier gas exchange, that is reduction in DL [10, 60, 61]. Furthermore the increase in small airways flow resistance, likely related to extravascular lung water accumulation, has been associated to the increase in systemic cytokine levels [58, 62]. In our study on ultratriathlon athletes a significant increase in ULCs, partially reduced after 12 hours, has been documented after exhaustive exercise. In parallel at the acute phase there was a significant decrease in spirometric indices of big and small airways flows and lung volume (remaining within the normal range values) and in ventilatory performance, and that result was also influenced by the inflammatory cascade [63].

## 6. Psychophysical Performances in Extreme Environment

Combination of mechanical, hemodynamic, biochemical, and hypoxemic mechanisms seems to be behind the PE in both hypoxic hypo- and hyperbaric conditions (high-altitude and breath-hold deep diving), leading to an increase in both lung vascular hydrostatic pressure (either heterogeneous or not) and permeability.

Breath-hold diving causes hyperbaric and hypoxic stress on the cardiopulmonary system [65, 66]. Peripheral “blood shift” into the intrathoracic cavity is a well-known physiological response which prevents chest wall from collapsing at depth, particularly at low lung volumes [67, 68]. Both the acute increase in pulmonary arterial pressure due to blood shift and the reduction in gas-lung volume with depth challenge the pulmonary capillary system. Increased intravascular pressure and especially transcapillary pressure may stretch the wall, weaken its integrity, and reduce natural vessel ability to sustain high mechanical stress [69]. A further increase in capillary pressure may cause the rupture of the alveolar-endothelial membrane, till leading to alveolar haemorrhage [8, 40, 70].

**Figure 2:**
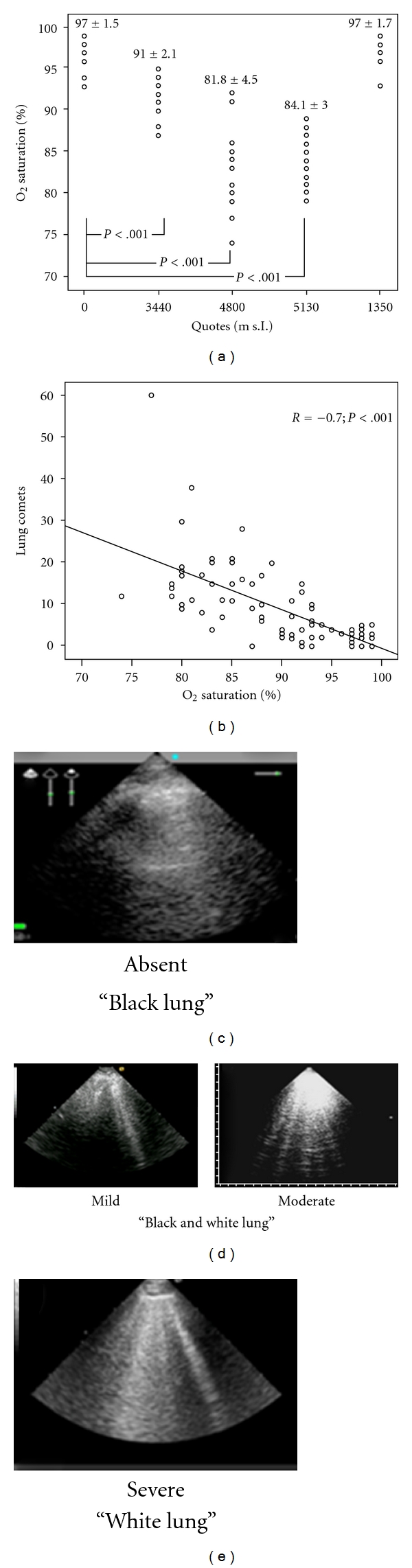
Progressive O_2_ saturation reduction at increasing altitude above sea level. Inverse correlation between O_2_ saturation and lung comets. Increase number of ULCs from sea level to 5130 meters above sea level (Pratali et al. [64]).

An additional potential mechanism responsible for such stress is the pulmonary vasoconstriction secondary to breath-holding hypoxia, with similarity to HAPE. Behind HAPE, physical exertion associated with hypoxic vasoconstriction may lead to an uneven redistribution of pulmonary capillary blood and ventilation, further leading to ventilo-perfusive mismatch that ultimately affects pulmonary gas exchange [21, 72]. West proposed that stress failure might be the initial factor for HAPE as seen in experimental animal models [73, 74]. According to this hypothesis recent studies have shown that the initial leak in HAPE is likely not inflammatory but related to increased hydrostatic pressure, with an alveolar increase in inflammatory mediators only at advanced stages, secondary to the high-pressure injury to the blood-gas barrier and/or PE formation [14]. In contrast, another study documented an increase in inflammatory mediators (IL-1B, IL-6, IL-8, and TNF-alpha) in broncoalveolar lavage fluid already at the early stage of HAPE [75]. In our study on recreational climbers, Chest ultrasonography revealed a high prevalence of clinically silent ULCs, that increased during ascent. In fact ULCs were present in 83% of subjects at 3440 m above sea level and in 100% of subjects at 4790 m above sea level, always with normal left and right ventricular function. Furthermore, ULCs were mirrored by decreased oxigen saturation (Figure 2), whereas no statistically significant correlation with systolic pulmonary arterial pressure rise during ascent was observed. Finally, ULCs decreased at descent [64].

Besides perplexities about mechanisms underlying PE in extreme environment and hypoxic conditions another problem is how early interstitial PE can be detected and measured. For example, there is the evidence that 92% of recreational climbers presenting chest rales or interstitial edema on the chest radiograph after ascent had increased closing volume. Furthermore 74% of climbers with no clinical or radiological evidence had probable subclinical edema, having an increase in closing volume at altitude (with no change in vital capacity) [4].

**Figure 3:**
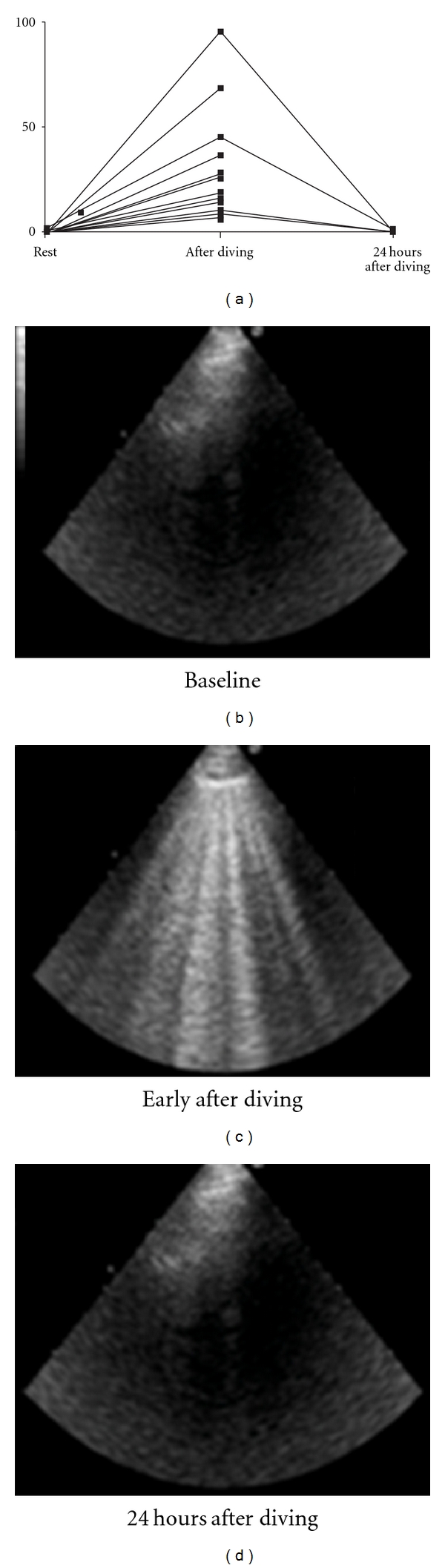
Chest ultrasonography revealed an increased number of ULCs within 10 minutes after immersion in 45% of 14 top-level breath-hold deep divers, which resolved completely after 24 hours (Frassi et al. [71]).

Chest ultrasonography revealed an increased number of ULCs within 10 minutes after immersion (depth range 31 to 112 meters) in 45% of 14 top-level breath-hold deep divers, which resolved completely after 24 hours (Figure 3). Of these, 2 had specific clinical symptoms, indicating a relatively high prevalence of subclinical extravascular lung water accumulation after breath hold deep diving [71]. In another study we measured DLCO to investigate the blood-gas barrier integrity after deep (30 meters) breath-hold diving at different time points (basely, within 2 minutes post dive, after 10 and 25 minutes). The early but transient increase in DLCO after diving in all subjects supported the hypothesis of intrathoracic capillary pooling of red blood cells (blood shift). Persistence at 25 minutes of high value of DLCO in one subject could be attributed to extravasation of blood into the alveoli by CT scan. In other 5 subjects, a late (at 25 minutes) decrease in DLCO more than 10% below the baseline, together with symptoms of dizziness in 3 of them, strongly suggested the presence of interstitial edema [3]. Furthermore we evaluated whether a hyperbaric stress condition associated to a prevalent hypoxic condition, as induced by a maximal static breath holding at 10 meters depth, may influence the integrity of the alveolar-capillary membrane and lung ventilo-perfusive heterogeneity. Accordingly we employed both DLCO and DLNO measurement basely, at 2-10-25 minutes after dive. We found an early (at 2 minutes) but transient increase in DLCO, likely indicating the persistence of capillary pooling of red blood cells following emersion (blood shift). This hypothesis was further supported by a parallel stable trend of DLNO, as expected being DLNO not affected by blood volume and flow. Finally DLNO significantly increased at 10 minutes likely secondary to pulmonary blood redistribution and thus probably reflecting a condition of ventilo-perfusive mismatch. Significant decrease of both DLNO and DLCO at 25 minutes strongly supported the hypothesis of alveolar-capillary membrane distress until interstitial fluid accumulation that is the condition affecting lung diffusive function [76]. 

## 7. Conclusions

Lung fluid accumulation in healthy subjects during the above-mentioned extreme conditions is a complex and multifactor phenomenon, still with unanswered questions. In particular whether the acute, reversible increase in lung fluid content is an innocent and benign part of the adaptation to extreme physiological condition or rather the clinically relevant marker of an individual vulnerability to life-threatening PE remains to be established in future studies. Thus the question if encouraging more conservative habits is right or not remains open. Chest ultrasonography is a low-cost highly feasible and versatile, nonionizing and noninvasive technique, with the undiscussed advantage to be used on the field early after exercise and repeated in the followup on a large sample of subjects. Further, chest ultrasonography has the potential to detect even subclinical episodes of lung fluid accumulation, thus it can provide new features on physiological mechanisms, incidence, and progression of this phenomenon with the final goal to identify vulnerable subjects.
